# Maternal Protein Restriction and Post-Weaning High-Fat Feeding Alter Plasma Amino Acid Profiles and Hepatic Gene Expression in Mice Offspring

**DOI:** 10.3390/foods11050753

**Published:** 2022-03-04

**Authors:** Moe Miyoshi, Kenji Saito, Huijuan Jia, Hisanori Kato

**Affiliations:** Health Nutrition, Department of Applied Biological Chemistry, Graduate School of Agricultural and Life Sciences, The University of Tokyo, 1-1-1 Yayoi, Bunkyo-ku, Tokyo 113-8657, Japan; monet344myo@gmail.com (M.M.); skkj774@gmail.com (K.S.); akatoq@g.ecc.u-tokyo.ac.jp (H.K.)

**Keywords:** maternal low-protein diet, high-fat diet, obesity, plasma amino acid, DNA microarray, hepatic gene expression

## Abstract

Maternal undernutrition during pregnancy is closely associated with epigenetic changes in the child, and it affects the development of obesity throughout the child’s life. Here, we investigate the effect of fetal low protein exposure and post-weaning high-fat consumption on plasma amino acid profiles and hepatic gene expression. Mother C57BL/6J mice were fed a 20% (CN) or 9% (LP) casein diet during pregnancy. After birth, the male offspring of both these groups were fed a high-fat diet (HF) from 6 to 32 weeks. At 32 weeks, the final body weight between the two groups remained unchanged, but the LP-HF group showed markedly higher white fat weight and plasma leptin levels. The LP-HF group exhibited a significant increase in the concentrations of isoleucine, leucine, histidine, phenylalanine, serine, and tyrosine. However, no differences were observed in the lipid content in the liver. According to the hepatic gene expression analysis, the LP-HF group significantly upregulated genes involved in the chromatin modification/organization pathways. Thus, maternal low protein and a post-weaning high-fat environment contributed to severe obesity states and changes in gene expression related to hepatic chromatin modification in offspring. These findings provide novel insights for the prevention of lifestyle-related diseases at the early life stage.

## 1. Introduction

Obesity is a major risk factor for many chronic diseases. Its development involves psychosocial stress, neuroendocrine regulation abnormalities, and genetic or epigenetic mechanisms. Maternal undernutrition during pregnancy and lactation changes the child’s epigenetic state; it is also one of the long-term risk factors for children’s obesity [1,2]. In particular, the more severe the mismatch between the environment adapted at the fetal stage and the environment encountered at the adult stage, the more serious the disease [3]. Several animal experiments have indicated that maternal undernutrition during pregnancy and high-fat feeding during post-weaning results in serious obesity when the offspring grow to become adults [4,5,6]. These studies investigated the effects of the interscapular brown adipose tissue [5], white adipose tissue [6], and the hypothalamus [4]. Moreover, low protein exposure during the fetal stage induces a preference for a high-fat diet (HFD) in young adult rats [7]. Therefore, it is important to clarify the mechanism by which maternal low-protein and offspring high-fat consumption increase the risk of obesity.

Some human and animal research has reported that changes in amino acid levels in blood and tissues are related to diseases such as obesity [8,9,10], diabetes [8,11], cardiovascular diseases [12], and cancer [13]. Amino acid profiles could serve as a biomarker of the propensity for these diseases. For instance, obesity and diabetes are related to the enhancement of branched-chain amino acid (BCAA) secretion in plasma, including leucine, isoleucine, and valine [9,14]. The plasma amino acid status of maternal protein-restricted mothers or offspring at weaning changes [15], but the plasma amino acid status of maternal protein-restricted adult offspring is still unclear.

The liver has gained increasing relevance in studies of obesity mechanisms owing to its role in glucose and lipid metabolism. Some of the alterations in the offspring liver, induced by maternal protein restriction, contribute to glucose [16,17] and lipid homeostasis [18,19]. Analysis of post-weaning rat offspring livers has shown that the hypomethylated state of the promoter region in the glucocorticoid receptor (GR) and peroxisomal proliferator-activated receptor alpha (PPARα) increases GR and PPARα mRNA expression through maternal protein restriction [20]. However, studies examining the effect of maternal low protein exposure using the liver often target the early life of the offspring; therefore, most studies have focused on the potential mechanisms before the onset of obesity. Maternal protein restriction and long post-weaning high-fat feeding conditions in offspring cause hepatic structural alterations [21] and sex-dependent hepatic gene expression changes related to glucose and fatty acid metabolism [22]; however, the effect on hepatic gene expression remains largely unknown.

The purpose of this study was to confirm the long-term effects of mismatch between maternal low-protein environment and prolonged HFD in adulthood by analyzing blood and liver samples. We fed a HFD to maternal protein-restricted mice offspring from 6–32 weeks. Using this mouse model, we assessed plasma and hepatic biochemical parameters, including plasma amino acid profiles. We also performed DNA microarray analysis using offspring livers to identify new characteristic pathways and gene alterations, and some genes identified in DNA microarray analysis were evaluated by real-time reverse-transcription polymerase chain reaction (RT-PCR).

## 2. Materials and Methods

### 2.1. Animals

The experiments were approved by and analyzed according to the guidelines of the Animal Usage Committee, Graduate School of Agricultural and Life Sciences, University of Tokyo. All mice were maintained at a temperature of 22 ± 1 °C and humidity of 60 ± 5% under a 12 h light–dark cycle. The mice were fed food and water ad libitum. After a 1-week acclimation, virgin C57BL/6J mice (8 weeks old) were mated to a single stud male. Pregnant dams were randomly allocated to be fed a 20% casein diet (CN) or 9% casein diet (LP) during pregnancy (*n* = 4, Table S1). After giving birth, the dams were fed an MF diet (a commonly used basal diet, Oriental Yeast, Tokyo, Japan), and the offspring were weaned at 4 weeks. Only male offspring were fed a HFD (45% fat calorie ratio) (Research Diets, New Brunswick, NJ, USA) for 6 weeks (maternal CN offspring as CN-HF group, *n* = 8; maternal LP offspring as LP-HF group, *n* = 9), and they were anatomized at 32 weeks. We collected the liver, kidney, plasma, gastrocnemius muscle, mesenteric fat, epididymal fat, and retroperitoneal fat.

### 2.2. Biochemical Test

All blood samples were collected in ethylenediamine-*N,N,N′,N′*-tetraacetic acid, dipotassium salt, and dehydrate (EDTA 2 K) containing tubes. The concentrations of the following biochemical markers were measured using commercial kits: glucose (Glu), triglyceride (TG), non-esterified fatty acid (NEFA), total cholesterol (TC), and high-density lipoprotein cholesterol (HDL-C) (Wako Pure Chemical Industries, Osaka, Japan); adiponectin (Otsuka Pharmaceutical, Tokyo); leptin and insulin (Morinaga Institute of Biological Science, Kanagawa, Japan); corticosterone (Assaypro, St. Charles, MO, USA); and insulin-like growth factor–1 (IGF-1) (R&D Systems, Minneapolis, MN, USA). Low-density lipoprotein cholesterol (LDL-C) was calculated as the concentration of TC, HDL-C, and TG (LDL-C = TC-HDL-C-TG/5).

For plasma amino acid analysis, plasma samples were mixed and centrifuged twice with 3% trichloroacetic acid solution. The supernatant was filtered through a 0.45 μm membrane. Plasma amino acid concentrations were measured using an automatic amino acid analyzer (L-8900; Hitachi, Tokyo, Japan). Type AN-2 and type B (Wako Pure Chemical Industries, Osaka, Japan) were used as the amino acid mixture standard solutions.

Total lipids in the liver were extracted using the Folch method, and the hepatic TG and TC concentrations were measured using commercial kits.

### 2.3. DNA Microarray Analysis

Total liver RNA was extracted using NucleoSpin^®^RNAII (Takara Bio, Siga, Japan). Individuals were randomly selected from each group (*n* = 3 in CN-HF; *n* = 4 in LP-HF). DNA microarray analysis was carried out using a Mouse Genome 430 2.0 Array Gene Chip (Affymetrix, Santa Clara, CA, USA). Data were normalized by robust multi-array average, and comparison of expression fluctuation between two groups was made using rank products. The probes were filtered under the condition of False Discovery Rate ≤ 0.2, and their functions were analyzed using the Database for Annotation, Visualization, and Integrated Discovery (DAVID Bioinformatics Resources 6.7).

### 2.4. Reverse-Transcription Polymerase Chain Reaction

We performed RT-PCR for RNA extracted from the liver using the same method as that used for DNA microarray analysis. The primer sequences are shown in Table S2. The relative amounts of mRNA were normalized to glyceraldehyde-3-phosphate dehydrogenase expression levels.

### 2.5. Statistical Analysis

Data are presented as mean ± standard error. Statistical significance was assessed using one-way analysis of variance with Student’s t-test. Statistical significance was set at *p* < 0.05.

## 3. Results

### 3.1. Changes in Body Weight, Food Intake, and Organ Weights

**Figure 1:**
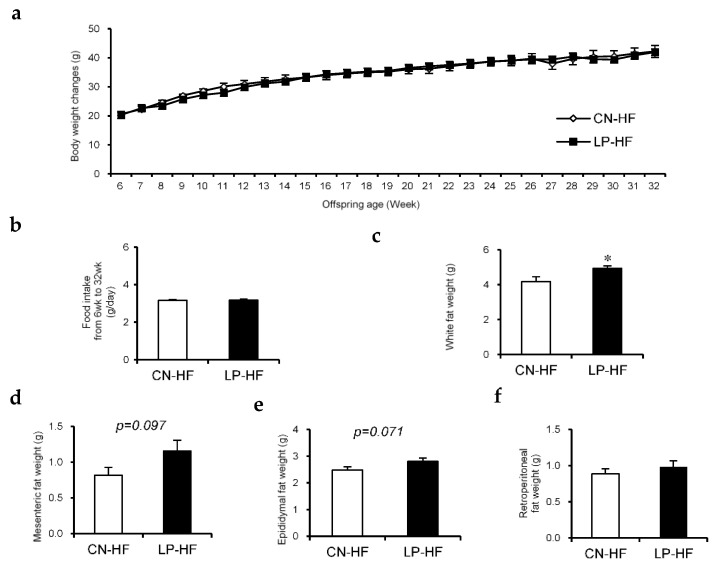
The effect of maternal low protein and post-weaning high-fat diet (HFD) intake on the offspring. (**a**) Body weight changes while the offspring were given HF diet; (**b**) Food intake from 6–32 weeks; (**c**) White fat weight, which contains mesenteric, epididymal, and retroperitoneal fat, at 32 weeks; (**d**) Mesenteric fat weight, (**e**) epididymal fat weight, and (**f**) retroperitoneal fat weight at 32 weeks. Measurement values were expressed as mean ± standard error (SE; *n* = 8/9). * *p* < 0.05 versus the CN-HF using Student’s *t*-test. CN-HF, maternal 20% casein offspring with HF group; LP-HF, maternal 9% casein offspring with HF group.

There were no significant differences in body weight changes and food intake between the two groups (Figure 1a,b). The weights of the liver, kidney, and gastrocnemius did not show any statistically significant changes (Table S3). However, the white fat weight, which includes mesenteric, epididymal, and retroperitoneal fat, in LP-HF was significantly increased compared to that in the CN-HF group (Figure 1c). In particular, the weights of mesenteric fat and epididymal fat tended to be higher in the LP-HF group (Figure 1d,e); however, the retroperitoneal fat weight did not differ between the two groups (Figure 1f).

### 3.2. Biochemical Markers in the Liver and Plasma

**Figure 2:**
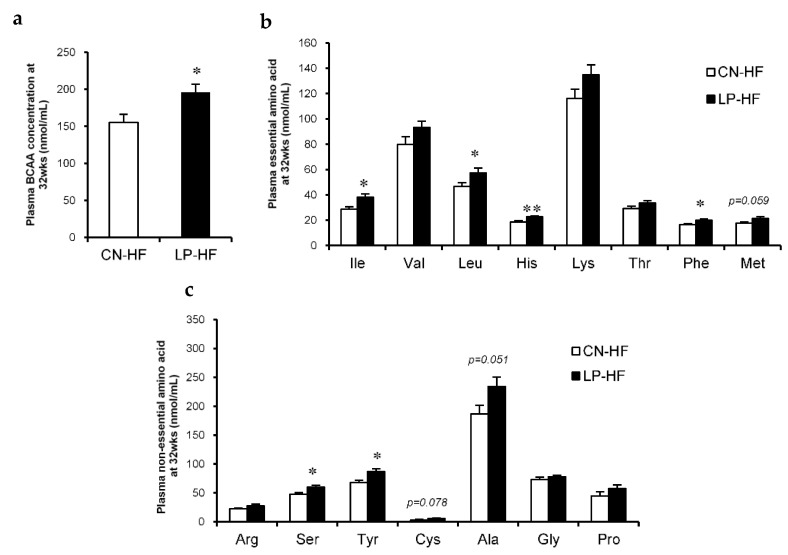
Plasma amino acid concentrations. (**a**) Plasma branched-chain amino acid (BCAA) concentration at 32 weeks; (**b**) Plasma essential amino acid concentrations at 32 weeks; (**c**) Plasma non-essential amino acid concentrations at 32 weeks. All values are mean ± standard error (SE; *n* = 8/9). * *p* < 0.05, ** *p* < 0.01 versus CN-HF using Student’s *t*-test. Isoleucine (Ile), Valine (Val), Leucine (Leu), Histidine (His), Lysine (Lys), Threonine (Thr), Phenylalanine (Phe), Methionine (Met), Arginine (Arg), Serine (Ser), Tyrosine (Tyr), Cysteine (Cys), Alanine (Ala), Glycine (Gly), Proline (Pro). BCAA, branched-chain amino acid. CN-HF, maternal 20% casein offspring with HF group; LP-HF, maternal 9% casein offspring with HF group.

With regard to the plasma biochemical markers, leptin levels were significantly increased in LP-HF; Glu and corticosterone levels in LP-HF tended to increase compared to those in CN-HF (Table 1). In addition, in LP-HF there were marked increases in the plasma BCAA levels (Figure 2a). We also observed that the concentrations of many plasma essential amino acids in LP-HF, such as isoleucine (Ile), leucine (Leu), histidine (His), and phenylalanine (Phe), were much higher than those in CN-HF (Figure 2b). Moreover, the concentrations of some plasma non-essential amino acids, including serine (Ser) and tyrosine (Tyr), also significantly increased in LP-HF compared to CN-HF (Figure 2c). The levels of plasma methionine (Met), cysteine (Cys), and alanine (Ala) tended to be higher in LP-HF than in CN-HF (Figure 2b,c). However, there were no significant changes in hepatic TC and TG levels between the two groups (Table 2).

### 3.3. Gene Expression Profiles in the Liver

We assessed the differences in hepatic gene expression profiles between CN-HF and LP-HF using DNA microarray analysis; the top four functional categories were lipid localization, chromatin modification, chromatin organization, and lipid transport (Table S4). The upregulated genes in LP-HF included several genes associated with lipid localization and transport, such as ATP-binding cassette, subfamily A, member 1 (*Abca1*), high-density lipoprotein binding protein (*Hdlbp*), oxysterol binding protein-like 9 (*Osbpl9*), phosphatidylinositol transfer protein, cytoplasmic 1 (*Pitpnc1*). In addition, genes related to chromatin modification and organization included chromodomain helicase DNA binding protein 4 (*Chd4*), lysine acetyltransferase 2 B (*Kat2b*), SWI/SNF-related, matrix-associated, actin-dependent regulator of chromatin, subfamily a, member 2 (*Smarca2*), SWI/SNF-related, matrix-associated, actin-dependent regulator of chromatin, subfamily c, member 1 (*Smarcc1*).

**Figure 3:**
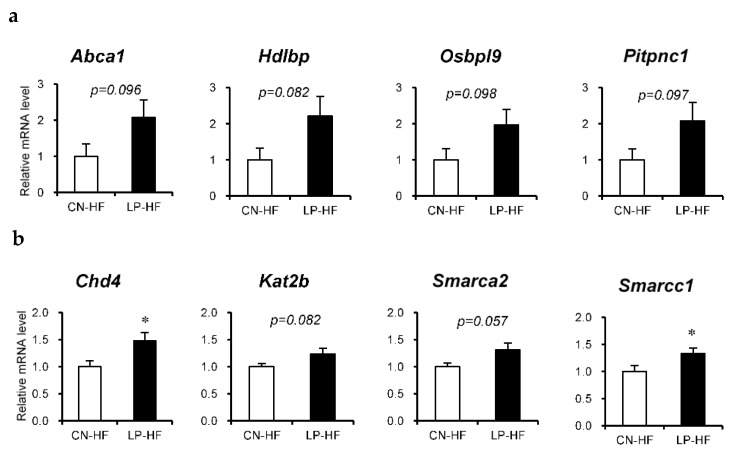
Relative mRNA expression in the liver. Genes that were found to be fluctuating in the DNA microarray were confirmed using real-time reverse-transcription polymerase chain reaction. (**a**) Genes related to lipid localization or lipid transport; (**b**) genes related to chromatin modification or chromatin organization. All values are mean ± standard error (SE; *n* = 8/9). * *p* < 0.05 versus CN-HF using Student’s *t*-test. CN-HF, maternal 20% casein offspring with high-fat diet group; LP-HF, maternal 9% casein offspring with HFD group.

Next, we assessed the relative mRNA expression of the eight selected genes using RT-PCR. The expression levels of *Chd4* and *Smarcc1* were significantly increased in LP-HF, and the expression levels of *Abca1*, *Hdlbp*, *Osbol9*, *Pitpnc1*, *Kat2b*, and *Smarca2* in LP-HF tended to be higher than those in CN-HF (Figure 3a,b).

## 4. Discussion

Many studies have proven that fetal undernutrition, followed by an HFD in adulthood, results in the development of obesity. Some have reported body weight gain and body fat accumulation in experimental animals [4,5]. In contrast, some animal studies have reported changes in biological parameters, gene expression, and protein levels, but no difference in body weight and white fat weight [6]. However, the types of obesity-related phenotypes appearing in animal experiments are inconsistent. In our study, we found no difference in final body weight, but a significantly increased white fat weight in LP-HF as compared to CN-HF. In addition, a significant increase in plasma leptin concentration was observed in LP-HF. Leptin, which is secreted by adipocytes, suppresses appetite and increases energy expenditure, mainly through the hypothalamus receptors. It is widely known that diet-induced obese mice have leptin resistance and exhibit hyperleptinemia proportional to body fat mass. In addition, offspring with fetal undernutrition show a premature leptin surge, which is believed to be one of the main causes of accelerated weight gain [4].

Plasma biochemical analysis also demonstrated an increase in several plasma amino acid levels in LP-HF, including Met, Cys, Ala, Ile, Leu, His, Phe, Ser, and Tyr. In particular, the elevation in plasma BCAA levels is a major characteristic of obesity, and it has been suggested that liver- and adipose tissue-specific alterations in BCAA metabolism contribute to this increase [9]. Moreover, some amino acids, such as Met, Ser, and Gly, are involved in epigenetic mechanisms by supplying methyl groups to DNA and histones via folate/methionine metabolism. Reportedly, a low-quality maternal protein diet, with 20% wheat gluten during gestation and lactation, lowers plasma Met concentrations in young rat offspring [15]. An interesting observation in our study was the increased plasma Met and Ser concentrations in LP-HF offspring. The relationship between plasma Met and Ser levels and epigenetic changes needs to be investigated in further studies.

Microarray analysis using hepatic mRNA identified that the top four differentiated pathways in LP-HF were lipid localization/transport and chromatin modification/organization. As for lipid localization/transport pathways, we focused on *Abca1*, *Hdlbp*, *Osbpl9*, and *Pitpnc1*. *Abca1* is a transporter central to the synthesis of HDL by mediating the efflux of cholesterol and phospholipids to lipoproteins [23], and low expression levels of *Abca1* reduce cholesterol flux into bile, as well as toward LDL [24]. *Hdlbp* affects the metabolism of major lipoproteins and specifically binds HDL molecules, which may function in the removal of excess cellular cholesterol [25]. *Osbpl9* and *Pitpnc1* are involved in lipid transport. The mRNA levels of these genes evaluated using RT-PCR showed an increasing trend in LP-HF, but there were no differences in lipid parameters in the blood and liver. Therefore, our data cannot explain how these genes affect the development of obesity risks in LP-HF offspring.

Regarding chromatin modification/organization pathways, we found that mRNA expression levels of *Chd4* and *Smarcc1*, which are involved in chromosome remodeling, were significantly upregulated in the LP-HF group. *Chd4* plays an important role in epigenetic transcriptional repression, as a main component of the nucleosome remodeling and deacetylase complex [26]. *Chd4* affects the DNA damage/repair network and is recruited to DNA-damaged sites in a poly (ADP-ribose) polymerase-dependent manner [27]. *Smarcc1*, a part of the SWI/SNF complex, is involved in epigenetic modifications [28], and is believed to regulate the transcription of certain genes by altering the chromatin structure around those genes using the energy from ATP hydrolysis [29]. Moreover, *Kat2b*, which is associated with histone acetylation, and *Smarca2*, which is involved in chromosome remodeling like *Smarcc1*, tended to have higher mRNA expression levels in LP-HF than in CN-HF. *Kat2b* stimulates the gluconeogenic program through a self-reinforcing cycle, whereby increases in the expression levels of H3K9Ac further potentiate the cyclic AMP response element-binding protein coactivator occupancy [30]. Notably, the increments of epigenetic modifier-relevant mRNA levels were observed in our results, because these genes catalyze the addition and/or removal of epigenetic signatures and contribute to gene expression regulation.

Further studies are needed to elucidate the relationship between our findings and epigenetic state alterations and to investigate the protein expression of the changed genes, which eventually influences function at the metabolic level. Studies are also needed to clarify the effect of a LP diet during lactation on the metabolic phenotype of offspring. Nevertheless, we expect that our study findings will be beneficial in demonstrating the potential of protein malnutrition during the early stages of life.

## 5. Conclusions

In conclusion, our findings suggest that HF-induced obesity symptoms become more severe in maternal protein-restricted offspring, and plasma biomarkers such as leptin and BCAAs strongly reflect an increased risk of obesity. The present study has demonstrated that there was a significant increase in plasma folate/methionine metabolism-relevant amino acid levels and hepatic epigenetic modifier-related gene expression, which may play an important role in the regulation of gene expression via epigenetic mechanisms. Our findings provide information on new therapeutic targets to prevent the onset of lifestyle-related diseases.

## Figures and Tables

**Table 1:** Plasma biochemical markers.

Plasma Concentration	CN-HF	LP-HF	*p* Value
Glu (mg/dL)	291.50 ± 20.38	348.62 ± 18.98	*p* = 0.058
TG (mg/dL)	73.25 ± 8.52	62.00 ± 6.60	
NEFA (mg/dL)	0.80 ± 0.07	0.72 ± 0.06	
TC (mg/dL)	160.66 ± 8.14	170.78 ± 10.19	
HDL-C (mg/dL)	109.91 ± 4.31	120.58 ± 4.82	
LDL-C (mg/dL)	36.10 ± 10.22	36.02 ± 14.08	
Adiponectin (ng/mL)	1.51 ± 0.06	1.56 ± 0.09	
Leptin (ng/mL)	27.67 ± 2.40	35.30 ± 1.75 *	
Insulin (ng/mL)	5.71 ± 1.50	4.23 ± 0.49	
Corticosterone (ng/mL)	1.02 ± 0.17	1.41 ± 0.13	*p* = 0.089
IGF-1 (pg/mL)	722.68 ± 40.46	721.55 ± 46.87	

All values are mean ± standard error (SE; *n* = 8/9). The *p* value is *p* < 0.1. * *p* < 0.05 versus CN-HF by Student’s *t*-test.

**Table 2:** Hepatic biochemical markers.

Concentration in the Liver	CN-HF	LP-HF
TG (mg/dL)	21.32 ± 3.63	22.55 ± 4.64
TC (mg/dL)	2.05 ± 0.16	2.26 ± 0.09

All values are mean ± standard error (SE; *n* = 8/9). No significant differences.

## Data Availability

Not applicable.

## Supplementary Materials

The following supporting information can be downloaded at: https://www.mdpi.com/article/10.3390/foods11050753/s1, Table S1: Composition of the diets given to mothers during pregnancy; Table S2: Primer sequences for RT-PCR; Table S3: Organ weights; Table S4: Function category of genes whose changes were observed in LP-HF by DNA microarray analysis.

## References

[1] Ford N.D., Patel S.A., Narayan K.M.V. (2017). Obesity in Low- and Middle-Income Countries: Burden, Drivers, and Emerging Challenges. Annu. Rev. Public Health.

[2] Chavatte-Palmer P., Tarrade A., Levy R. (2012). Developmental origins of health and disease in adults: Role of maternal environment. Gynecol. Obstet. Fertil..

[3] Gluckman P.D., Hanson M., Spencer H. (2005). Predictive adaptive responses and human evolution. Trends Ecol. Evol..

[4] Yura S., Itoh H., Sagawa N., Yamamoto H., Masuzaki H., Nakao K., Kawamura M., Takemura M., Kakui K., Ogawa Y. (2005). Role of premature leptin surge in obesity resulting from intrauterine undernutrition. Cell Metab..

[5] Sellayah D., Dib L., Anthony F.W., Watkins A.J., Fleming T.P., Hanson M.A., Cagampang F.R. (2014). Effect of maternal protein restriction during pregnancy and postweaning high-fat feeding on diet-induced thermogenesis in adult mouse offspring. Eur. J. Nutr..

[6] Lukaszewski M.-A., Mayeur S., Fajardy I., Delahaye F., Dutriez-Casteloot I., Montel V., Dickes-Coopman A., Laborie C., Lesage J., Vieau D. (2011). Maternal prenatal undernutrition programs adipose tissue gene expression in adult male rat offspring under high-fat diet. Am. J. Physiol. Metab..

[7] Bellinger L., Lilley C., Langley-Evans S.C. (2004). Prenatal exposure to a maternal low-protein diet programmes a preference for high-fat foods in the young adult rat. Br. J. Nutr..

[8] Zhou Y., Qiu L., Xiao Q., Wang Y., Meng X., Xu R., Wang S., Na R. (2013). Obesity and diabetes related plasma amino acid alterations. Clin. Biochem..

[9] She P., Van Horn C., Reid T., Hutson S.M., Cooney R.N., Lynch C.J. (2007). Obesity-related elevations in plasma leucine are associated with alterations in enzymes involved in branched-chain amino acid metabolism. Am. J. Physiol. Metab..

[10] Horakova O., Hansikova J., Bardova K., Gardlo A., Rombaldova M., Kuda O., Rossmeisl M., Kopecký J. (2016). Plasma Acylcarnitines and Amino Acid Levels As an Early Complex Biomarker of Propensity to High-Fat Diet-Induced Obesity in Mice. PLoS ONE.

[11] Wang T.J., Larson M., Vasan R.S., Cheng S., Rhee E.P., McCabe E., Lewis G.D., Fox C.S., Jacques P.F., Fernandez C. (2011). Metabolite profiles and the risk of developing diabetes. Nat. Med..

[12] Ruiz-Canela M., Toledo E., Clish C., Hruby A., Liang L., Salas-Salvadó J., Razquin C., Corella D., Estruch R., Ros E. (2016). Plasma Branched-Chain Amino Acids and Incident Cardiovascular Disease in the PREDIMED Trial. Clin. Chem..

[13] Zhang L., Han J. (2017). Branched-chain amino acid transaminase 1 (BCAT1) promotes the growth of breast cancer cells through improving mTOR-mediated mitochondrial biogenesis and function. Biochem. Biophys. Res. Commun..

[14] Newgard C.B., An J., Bain J.R., Muehlbauer M.J., Stevens R.D., Lien L.F., Haqq A.M., Shah S.H., Arlotto M., Slentz C.A. (2009). A branched-chain amino acid-related metabolic signature that differentiates obese and lean humans and contributes to insulin resistance. Cell Metab..

[15] Cetin A.K., Dasgin H., Gülec A., Onbasilar I., Akyol A. (2015). Maternal Low Quality Protein Diet Alters Plasma Amino Acid Concentrations of Weaning Rats. Nutrients.

[16] Vo T.X., Revesz A., Sohi G., Ma N., Hardy D.B. (2013). Maternal protein restriction leads to enhanced hepatic gluconeogenic gene expression in adult male rat offspring due to impaired expression of the liver X receptor. J. Endocrinol..

[17] Zheng J., Xiao X., Zhang Q., Yu M., Xu J., Wang Z. (2014). Maternal protein restriction induces early-onset glucose intolerance and alters hepatic genes expression in the peroxisome proliferator-activated receptor pathway in offspring. J. Diabetes Investig..

[18] Torres N., Bautista C.J., Tovar A.R., Ordáz G., Rodríguez-Cruz M., Ortiz V., Granados O., Nathanielsz P.W., Larrea F., Zambrano E. (2010). Protein restriction during pregnancy affects maternal liver lipid metabolism and fetal brain lipid composition in the rat. Am. J. Physiol. Metab..

[19] Ramadan W.S., Alshiraihi I., Al-Karim S. (2013). Effect of maternal low protein diet during pregnancy on the fetal liver of rats. Ann. Anat.-Anat. Anz..

[20] Lillycrop K., Phillips E.S., Jackson A.A., Hanson M., Burdge G.C. (2005). Dietary Protein Restriction of Pregnant Rats Induces and Folic Acid Supplementation Prevents Epigenetic Modification of Hepatic Gene Expression in the Offspring. J. Nutr..

[21] Souza-Mello V., Mandarim-De-Lacerda C.A., Aguila M.B. (2007). Hepatic structural alteration in adult programmed offspring (severe maternal protein restriction) is aggravated by post-weaning high-fat diet. Br. J. Nutr..

[22] van Straten E.M.E., Bloks V.W., van Dijk T.H., Baller J.F.W., Huijkman N.C.A., Kuipers I., Verkade H.J., Plösch T. (2012). Sex-dependent programming of glucose and fatty acid metabolism in mouse offspring by maternal protein restriction. Gend. Med..

[23] Feingold K.R. (2000). Introduction to Lipids and Lipoproteins. Endotext.

[24] Szántó M., Gupte R., Kraus W.L., Pacher P., Bai P. (2021). PARPs in lipid metabolism and related diseases. Prog. Lipid Res..

[25] Oliva J., French S.W., Li J., Bardag-Gorce F. (2012). Proteasome inhibitor treatment reduced fatty acid, triacylglycerol and cholesterol synthesis. Exp. Mol. Pathol..

[26] O’Shaughnessy A., Hendrich B. (2013). CHD4 in the DNA-damage response and cell cycle progression: Not so NuRDy now. Biochem. Soc. Trans..

[27] Nio K., Yamashita T., Okada H., Kondo M., Hayashi T., Hara Y., Nomura Y., Zeng S.S., Yoshida M., Hayashi T. (2015). Defeating EpCAM+ liver cancer stem cells by targeting chromatin remodeling enzyme CHD4 in human hepatocellular carcinoma. J. Hepatol..

[28] Marei H.E., Ahmed A.-E. (2013). Transcription factors expressed in embryonic and adult olfactory bulb neural stem cells reveal distinct proliferation, differentiation and epigenetic control. Genomics.

[29] Peterson C., Tamkun J. (1995). The SWI-SNF complex: A chromatin remodeling machine?. Trends Biochem. Sci..

[30] Ravnskjaer K., Hogan M.F., Lackey D., Tora L., Dent S.Y., Olefsky J., Montminy M. (2013). Glucagon regulates gluconeogenesis through KAT2B- and WDR5-mediated epigenetic effects. J. Clin. Investig..

