# Supplementary material for: Maternal Protein Restriction and Post-Weaning High-Fat Feeding Alter Plasma Amino Acid Profiles and Hepatic Gene Expression in Mice Offspring
Source: Foods. 2022 Mar 4;11(5):753. doi: 10.3390/foods11050753 (PMC8909731; doi:10.3390/foods11050753)
Supplement: Supplementary file 1 [file foods-11-00753-s001.zip › foods-1599583 Supplementary.pdf]

Maternal protein restriction and postweaning high-fat feeding
alters plasma amino acid profiles and hepatic gene expression in mice offspring

(Moe Miyoshi, Kenji Saito, Huijuan Jia, Hisanori Kato)

Supplementary Materials and Methods

Table S1. Composition of the diets given to mothers during pregnancy.

Component (%)	CN (20% Casein)	LP (9% Casein)
Casein	20.0	9.0
Corn starch	66.8	77.8
DL-Methionine	0.2	0.2
Soy bean oil	5.0	5.0
Vitamin mixture *	1.0	1.0
Mineral mixture *	4.0	4.0
Cellulose powder	3.0	3.0

* AIN-76 prescription (Oriental Yeast, Tokyo, Japan).

Table S2. Primer sequences for RT-PCR.

Gene symbol	Primer sequences
Abca1	Forward: 5'-GGCGGCAACAAACGAAA-3'
	Reverse: 5'-AATGCTTAGGGCACAATTCCAC-3'
Hdlbp	Forward: 5'-CCGCTTGGAACATGAAGTGAA-3'
	Reverse: 5'-CATACCCTGTGATGGTGATTTGG-3'
Osbpl9	Forward: 5'-AATTGAAGGGGAATGGAATGG-3'
	Reverse: 5'-AAAGGCTGCGGGACTCATAC-3'
Pitpnc1	Forward: 5'-TTCAGACCCGAGTGGAACAA-3'
	Reverse: 5'-ACTCATCAACCCACGCAAAG-3'
Chd4	Forward: 5'-TGAAGAAAGAAGCATCGGTGAA-3'
	Reverse: 5'-GCCCAGTCAATAGAACCCAAGA-3'
Kat2b	Forward: 5'-GTCTACCCTG GACTTTCGTG TTTC-3'
	Reverse: 5'-TGGGCTCTTT ACTTTTCTCT TTTCC-3'
Smarca2	Forward: 5'-GAAGAGGAAGAAGATGATGAAGAGG-3'
	Reverse: 5'-CGACGGGTTTGGCTTTG-3'
Smarcc1	Forward: 5'-GCCGGGAAGAAGAAAGTGG-3'
	Reverse: 5'-TCAACCAAGAGAGCTACCAAGGA-3'
Gapdh	Forward: 5'-GGCACAGTCAAGGCTGAGAATG-3'
	Reverse: 5'-ATGGTGGTGAAGACGCCAGTA-3'

(Moe Miyoshi, Kenji Saito, Huijuan Jia, Hisanori Kato)

Supplementary Results

Table S3. Organ weights.

Organ Weight (g)	32wk	
	CN-HF	LP-HF
Liver	1.41 ± 0.09	1.69 ± 0.15
Kidney	0.42 ± 0.01	0.40 ± 0.01
Muscule (gastrocnemius)	0.28 ± 0.01	0.29 ± 0.004
Mesenteric fat	0.82 ± 0.11	1.15 ± 0.15
Epididymal fat	2.48 ± 0.12	2.80 ± 0.12
Retroperitonal fat	0.89 ± 0.07	0.98 ± 0.09

All values are mean ± SE (n = 8).

Table S4. Function category of genes whose changes were observed in LP-HF by DNA microarray analysis.

Probes with FDR ≤ 0.2 were extracted and uploaded to DAVID (n = 3–4).

The following are functional categories in which fluctuations were observed in Gene Ontology BP.

**Terms**	**p-Value**
lipid localization	5.5E-04
chromatin modification	6.6E-04
chromatin organization	6.9E-04
lipid transport	1.7E-03
steroid metabolic process	2.4E-03
cholesterol metabolic process	3.4E-03
transcription	4.7E-03
sterol metabolic process	5.2E-03
chromosome organization	6.1E-03
